# Assessment of Impact of Aramid Fibre Addition on the Mechanical Properties of Selected Asphalt Mixtures

**DOI:** 10.3390/ma13153302

**Published:** 2020-07-24

**Authors:** Damian Wiśniewski, Mieczysław Słowik, Jan Kempa, Agnieszka Lewandowska, Joanna Malinowska

**Affiliations:** 1 Faculty of Civil and Environmental Engineering and Architecture, UTP University of Science and Technology in Bydgoszcz, Prof. S. Kaliskiego 7, 85-796 Bydgoszcz, Poland; janke@utp.edu.pl; 2 Institute of Civil Engineering, Faculty of Civil and Transport Engineering, Poznan University of Technology, Piotrowo 5, 61-138 Poznań, Poland; mieczyslaw.slowik@put.poznan.pl; 3 TORMEL Sp. z o.o., Poznańska 105, 62-052 Komorniki, Poland; lewandowskaagnieszka9@gmail.com; 4 Zarząd Dróg Wojewódzkich w Bydgoszczy, Dworcowa 80, 85-010 Bydgoszcz, Poland; joannamalinowska160@gmail.com

**Keywords:** aramid fibre, rutting, indirect tensile strength ratio (*ITSR*), stiffness modulus, hot mix asphalt

## Abstract

Various additives to asphalt binders and asphalt mixtures improving their properties are being used more and more frequently in order to improve the durability of road pavements. Such additives include various types of fibres, including aramid fibres. Tests concerning the impact of aramid fibre addition on the properties of selected asphalt mixtures have been described herein. Two types of asphalt mixtures were assessed: high modulus asphalt concrete (HMAC) and stone mastic asphalt (SMA). The examined asphalt mixtures were assessed with regard to: resistance to rutting, resistance to water and frost as well as fatigue resistance. The conducted tests showed relatively small impact of aramid fibre addition on the improvement of some assessed properties of the analysed asphalt mixtures. The obtained results were also compared to results of the tests conducted by the other research team concerning the impact of aramid fibre addition on the properties of the other types of asphalt mixtures.

## 1. Introduction

Road pavement may be characterised using the following properties: bearing capacity, longitudinal and transverse evenness, skid resistance and a surface condition, such as: microtexture, macrotexture, losses of grains or asphalt binder, potholes and patches. The most significant factor that determines the quality and durability of pavement, which influences its technical condition as well, is an asphalt mixture (HMA—hot mix asphalt), used to make the upper layers of the pavement (wearing course, binder course and base course).

An asphalt mixture should be characterised by good functional properties already in the course of its designing, such as: high resistance to rutting, water and frost as well as fatigue resistance. These properties enable the fulfilment of specific requirements resulting in reducing pavement degradation and consequently improving a pavement condition and provide a longer service life. As early as in the course of designing an asphalt mixture, it is desirable for HMA to be of high stiffness modulus at high temperatures, which should improve its resistance to rutting. On the other hand, low values of stiffness modulus are desirable for improving low-temperature crack resistance. Various additives modifying an asphalt binder (a ‘wet’ method) as well as asphalt mixture (a ‘dry’ method) are used in order to achieve such properties.

Polymers (divided into elastomers and plastomers) are among the most popular additives used in asphalt binder modifications affecting properties of produced HMA. Plastomers increase the values of stiffness modulus of binders at high temperatures, without improving low-temperature properties. On the other hand, elastomer modified binders are characterised by a wide range of temperatures with viscoelastic behaviour that improve asphalt pavement resistance to permanent deformations, fatigue cracking and low-temperature cracking. The greatest advantage of using elastomers as bitumen modifiers is a significant improvement of asphalt binder elasticity [1,2,3], which results in the fact that elastomers are the most often used polymers for binder modification. Styrene-butadiene-styrene copolymer (SBS) is the most frequently used elastomer additive to asphalt binders.

The effect of SBS copolymer addition on asphalt properties has been described in various publications [1,4]. Mielczarek et al. [4] presented the results of the investigation concerning the impact of content of SBS copolymer addition on the properties of asphalt binders. Based on the obtained results, an improvement in the functional properties of road pavements with the use of asphalt binders modified with SBS copolymer was observed, in consequence increasing the durability of the pavement. Studies have shown an increase in dynamic shear modulus |G*| and a decrease in the phase angle values as the SBS copolymer content increases. This might indicate an increase in road resistance to rutting (permanent deformation). The SBS copolymer also reduced the susceptibility of the asphalt binder to temperature changes [4].

Not only polymers are used to modify asphalt binders and mixtures. Researchers also dealt with other additives, as indicated in the literature, including crumb rubber [5,6,7,8,9] produced as a result of waste tyre processing as well as natural asphalts [10,11,12].

Various types of fibres that, like other additives, may improve HMA properties, are becoming more frequent as additives to asphalt mixtures. The addition of randomly oriented fibres to an asphalt mixture may improve its mechanical properties that consequently result in the improvement of road pavement durability. Fibres intended for asphalt mixture reinforcement may be natural fibres such as: coconut, sisal, hemp or jute [13]. Additionally, polypropylene, polyester asbestos, cellulose, coal, glass and nylon fibres [14] are used as modifiers. Research works indicate that fibre addition to an asphalt mixture results in the improvement of rutting resistance [15,16,17,18], the increase of HMA stiffness modulus [15,17], the increase of tensile strength [15,19,20], and the increase of stability determined according to the Marshall method [17,18,20]. Moreover, tests indicate a possibility of fatigue resistance improvement of HMA [15,16,17,18]. Some sources indicate that fatigue resistance improvement may be achieved only with respect to moderate and small deformations [15,19], which may imply that an asphalt mixture modified in this manner will be an appropriate solution with regard to fatigue resistance of the pavements subjected to high-speed traffic (including motorways) [15]. As a result of fibre addition to HMA, its resistance to low-temperature cracking may increase, which has been confirmed in field conditions on a selected pavement section (an analysis period was one-year long—including two summer periods). When it comes to a pavement made of an asphalt mixture without fibres, a few small cracks were found, whereas on a pavement made of a mixture with fibre addition, no cracks were detected [15].

An additive used for HMA can also be basalt fibres, which improve HMA properties, such as dynamic stability, tensile stress or TSR, which Guo presents in his work [21]. The positive effect of the addition of basalt fibres is also confirmed by Xiao Qin, who in his work [22] presented the improvement of asphalt mastics resistance to cracking.

A solution of the so-called hybrid reinforcement of HMA using fibres may be found in the analysis of relevant literature. An example is a study [23] in which the addition of glass fibre using the dry method was applied to an aggregate mixture as well as addition of polypropylene fibres using the wet method (to asphalt binder). Polypropylene fibres at 150 °C become viscous, which makes it possible for them to combine homogenously with an asphalt binder. The binder after combining with fibres was characterised by lower penetration and ductility, as well as a higher softening point value. Asphalt binder modified as above and aggregate combined with glass fibre were used to produce an asphalt mixture. HMA prepared in such a way was characterised by increased value of the Marshall stability and decreased value of the Marshall flow.

Aramid fibres are used to modify HMA as well. Those fibres belong to the group of aromatic polyamides. They are built of linear, regular, and rigid chains of para-phenylene terephthalamide particles. They are recognised as synthetic fibres characterised by high stiffness modulus. They are combined with strong and dense hydrogen bonds, thanks to which they achieve good mechanical properties. Aramid fibres are characterised by a favourable strength to density ratio. Their density is 43% lower compared to glass fibres. Moreover, they are characterised by good properties within fatigue resistance, wearing resistance, high fracture energy as well as a low thermal expansion coefficient [24]. The addition of aramid fibres has a significant influence on the improvement of asphalt mixture properties. According to conducted tests [23,25,26], it has an impact on indirect tensile strength, improvement of rutting and fatigue resistance as well as resistance to low temperature cracking. It may, however, decrease HMA water and frost resistance as it reduces an indirect tensile strength ratio value compared to an asphalt mixture without aramid fibre addition [19].

Angel Mateos and John Harvey prepared a report about the effect of the addition of aramid fibres on the properties of HMA used in California [27], which also indicate their positive effect. The addition of fibres to HMA results in improved the fatigue life at high strain levels (at 600–900 μm/m), which cannot be observed for medium strain levels. The fibres improved HMA rutting resistance, which was confirmed by tests performed using asphalt mixture performance tester (AMPT).

In literature, we can find studies of very short aramid fibres, which include Aramid Pulp Fibres. Saliani and Badeli conducted such research and described the influence of the addition of these fibres on HMA properties in their works [28,29]. The research they carried out shows that the addition of these fibres increase the durability against freeze-thaw cycles and increase the HMA resistance to low temperature cracking. The addition of these fibres to hot mix asphalt also allowed for better ductibility, which, according to the authors of the above papers, can improve crack resistance at low temperatures.

Aramid fibres can be combined with other types of fibres, which allows one material to be obtained. Polyolefin-aramid fibres can be such an example. Ziari in his paper [30] presented the results showed that this addition has a positive effect on rutting resistance, cracking performance and fatigue performance. Slebi [31] also shows in his research that the addition of 0.3% polyolefin-aramid fibres significantly improves HMA endurance at low temperature (−15 °C).

The motivation for undertaking the research presented in this article were promising research results of the impact of the aramid fibres addition on the properties of asphalt mixtures obtained by researchers at the Gdańsk University of Technology. The aim of the study was to assess the impact of aramid fibres addition on the properties of asphalt mixtures other than those selected by researchers from Gdańsk, which are also used for road construction in Poland.

## 2. Materials and Methods

**Figure 1:**
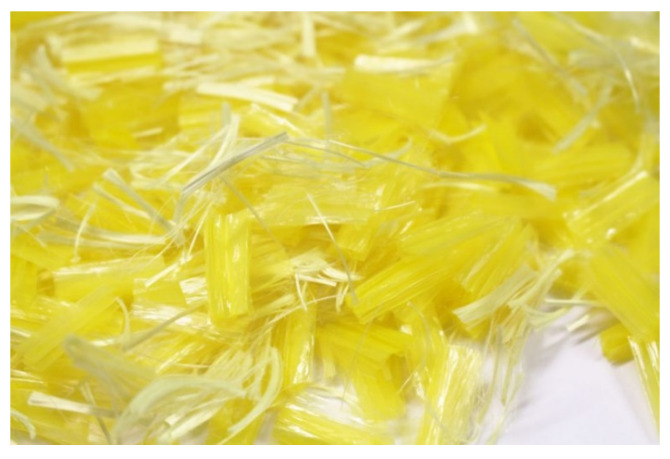
The aramid fibres Forta-fi [32].

Two types of asphalt mixtures were tested: HMAC 16B 20/30 (high modulus asphalt concrete—HMAC with a maximum particle size of 16 mm intended for a heavy load pavement binder course, including 20/30 penetration grade bitumen) and SMA 11 PMB 45/80-55 (stone mastic asphalt with a maximum aggregate particle size of 11 mm, including polymer modified bitumen PMB 45/80-55, intended for heavy load traffic pavement wearing course). Each of the analysed asphalt mixtures was produced in two versions: one without aramid fibres (a reference mixture) and one with aramid fibre addition in the quantity of 0.5 kg of aramid fibres for 1000 kg of HMA, which constitutes 0.05% m/m (being the quantity recommended by the producer). Forta-fi aramid fibres were used for the research as an additive to asphalt mixtures. They were characterized by the following parameters: length 19 mm, density 1.44 Mg/m^3^, tensile strength 2.76 GPa. The fibres were added directly to the mixer at the stage of mixing the components of the mineral mix even before the asphalt binder was added. The process of mixing the ingredients was carried out in the same way as for the mixture without the addition of fibres. The aramid fibres used for the tests are shown in Figure 1.

Coarse and fine amphibolitic aggregate was used to produce the analysed asphalt mixtures. They were manufactured using two types of asphalt binders −20/30 penetration grade bitumen as well as polymer modified bitumen PMB 45/80-55. Road bitumen 20/30 is the hardest asphalt binder (Orlen Asfalt, Płock, Poland) produced in Poland. Due to its high softening point value and high sensitivity to low-temperature cracking, its usage is recommended only to produce high modulus asphalt concrete (HMAC) paved in binder and base courses. Polymer modified bitumen PMB 45/80-55 is an asphalt binder modified by a polymer which is most frequently used in asphalt mixtures intended for wearing courses of road pavements.

Grading and dust content in particular fractions of aggregate had been determined before tests, in accordance with the EN 933-1:2012 standard. Moreover, basic bitumen parameters had been indicated—penetration at 25 °C (according to the EN 1426:2015-08 standard) and a softening point using the Ring and Ball method (according to the EN 1427:2018-08 standard). Results of bitumen tests have been presented in Table 1.

**Figure 2:**
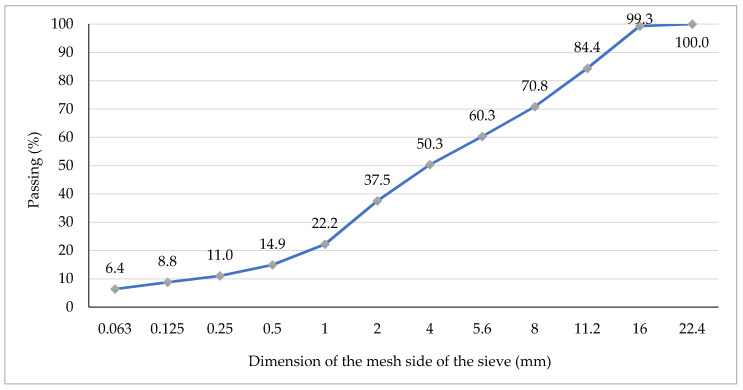
Graph of grain size distribution curve of the HMAC 16B mixture.

**Figure 3:**
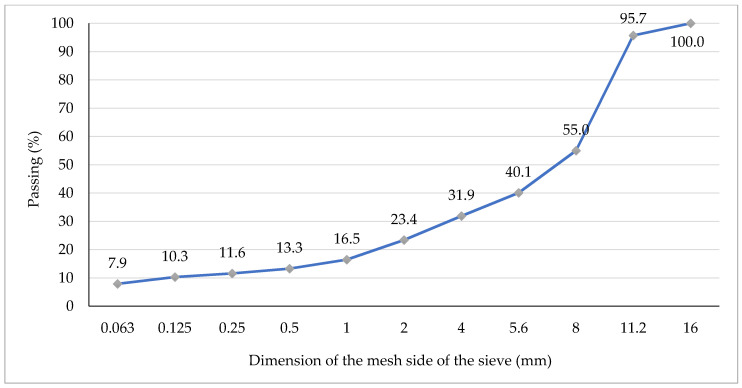
Graph of grain size distribution curve of the SMA11 mixture.

Analysed asphalt mixtures were prepared in laboratory conditions in accordance with the EN 12697-35:2016-05 standard. Content of all components used in the mineral aggregate mixture (AM) and the hot mix asphalt (HMA) of HMAC 16B have been presented in Table 2, whereas components of stone mastic asphalt SMA 11 have been presented in Table 3. Additionally, graphs of grain size distribution curves for the analysed asphalt mixtures: HMAC 16B and SMA11 are presented in Figure 2 and Figure 3, respectively.

Viatop stabilizer was added to the SMA 11 asphalt mixtures (with and without fibres) in the amount of 0.4% by weight of the HMA. It is a granulate made of modified cellulose fibres. Its function is to stabilize and reinforce the hot mix asphalt. Viatop fibres prevent the flow down of asphalt binder from the aggregate’s surface.

Results of rutting, water and frost resistance tests, their stiffness modulus and fatigue resistance tests of the analysed asphalt mixtures have been presented and described herein. Some of these results were compared to the results obtained by researchers from the Gdańsk University of Technology, who assessed the effect of the aramid fibres addition on the following hot mix asphalt types properties: AC16B 35/50 (asphalt concrete with a maximum aggregate grain size of 16 mm intended for a binder course, containing 35/50 penetration grade bitumen), AC16B PMB 25/55-60 (asphalt concrete with a maximum aggregate grain size of 16 mm intended for a binder course, containing polymer modified bitumen PMB 25/55-60) and AC11W 50/70 (asphalt concrete with a maximum aggregate grain size of 11 mm intended for a wearing course, containing 50/70 penetration grade bitumen) [26].

### 2.1. Determination of Air Void Content of Asphalt Mixtures

Determination of air void content was carried out in accordance with EN 12697-8:2018 on the basis of density and bulk density. The density was determined using a pycnometer in accordance with EN 12697-5:2018. The bulk density was determined by method B according to EN 12697-6:2012. The content of air voids was determined on the basis of Equation (1):

(1) $$V_{m}=\frac{\left(\rho_{m}-\rho_{b}\right)}{\rho_{m}}\;\;\;\;\left(\%\right)$$

where:

$V_{m}$—content of voids in the hot mix asphalt (%);

$\rho_{m}$—density of the hot mix asphalt [g/cm^3^];

$\rho_{b}$—bulk density of the hot mix asphalt [g/cm^3^].

### 2.2. Tests of Resistance to Permanent Deformation of Asphalt Mixtures

Resistance to permanent deformation is one of the main requirements for asphalt mixtures used in road and airport pavements. It enables to specify its susceptibility to permanent deformation caused by moving vehicle wheel load. Rutting resistance may be examined by measuring the depth of a rut formed as a result of the repeated passage of a loaded wheel at a given temperature. The test was conducted in accordance with the EN 12697-22:2008 standard using a wheel tracker (small device). It was performed according to B procedure—in air, at temperature of 60 °C, using 10,000 load cycles. Samples of the asphalt mixture were compacted using the roller compactor device according to the EN 12697-33:2019-03 standard. The dimensions of the samples were 500 × 800 mm. Thickness of the prepared samples depended on the maximum size of the aggregate grains contained in the hot mix asphalt and was equal to 41 mm for SMA 11 and 62 mm for HMAC 16B. Results of measurements are described using the following parameters: a wheel-tracking slope determined with Equation (2) and an average proportional rut depth determined using Equation (3):

(2) $$WTS_{\mathrm{AIR}}=\frac{\left(d_{10000}-d_{5000}\right)}{5}\;\;\;\;\left(\frac{mm}{10^{3}load\;cycles}\right)$$

where:

$WTS_{\mathrm{AIR}}$—a wheel-tracking slope (mm/10^3^ load cycles);

$d_{5000}$—rut depth after 5000 load cycles (mm);

$d_{10000}$—rut depth after 10,000 load cycles (mm).

(3) $$PRD_{\mathrm{AIR}}=\frac{d_{10000}}{h}\cdot 100\left(\%\right)\;$$

where:

$PRD_{\mathrm{AIR}}$—proportional rut depth (%);

$d_{10000}$—rut depth after 10,000 load cycles (mm);

h—height of an asphalt mixture sample subjected to rutting test (mm).

### 2.3. Test of Resistance to Water and Frost of Asphalt Mixtures

The test was performed according to the EN 12697-12:2018-08 standard and in accordance with the procedure described in the Polish Technical Requirements WT-2:2014 (part I). In order to determine the resistance to water and frost, 10 samples of hot mix asphalt compacted using a Marshall compactor (infraTest, Berlin, Germany) were prepared, in accordance with EN 12697-30:2012, using 35 impacts on each side of the sample. The height of compacted samples should be 63.5 ± 2.5 mm, while their diameter 101.6 ± 0.1 mm. After the samples were compacted, their dimensions were measured in accordance with EN 12697-29:2003 and bulk density in accordance with EN 12697-6:2012. In the next step, the prepared samples were divided into two sets: “dry” set (without conditioning) and “wet” set (with conditioning). Each set consisted of five samples. The division was made in such a way that both sets had as close as possible average heights and values of bulk density. Dry set samples were not conditioned; they were stored at room temperature (20 ± 5 °C). The wet set samples are conditioned by placing them in a vacuum apparatus for soaking with water (the degree of water saturation must be between 55% and 80%). The samples were then placed in a water bath at 40 ± 1 °C for 68–72 h. In the next step, the samples were subjected to freezing for a minimum of 16 h at (−18 ± 3 °C), after which they were placed in a water bath at 25 ± 2 °C for a further period of 24 ± 1 h.

Resistance to water and frost of an asphalt mixture was determined on the basis of an indirect tensile strength ratio (*ITSR*). This parameter was calculated as a ratio of indirect tensile strength determined on ‘wet’ samples (after conditioning) to indirect tensile strength determined on ‘dry’ samples (without conditioning), using the following equation:

(4) $$ITSR=\frac{ITS_{w}}{ITS_{d}}\cdot 100\left(\%\right)$$

where:

ITSR—indirect tensile strength ratio (%);

$ITS_{w}—$average value of indirect tensile strength of ‘wet set’ samples (kPa);

$ITS_{d}—$average value of indirect tensile strength of ‘dry set’ samples (kPa).

### 2.4. Stiffness Modulus Determination of Asphalt Mixtures

Stiffness modulus determination was conducted in accordance with the EN 12697-26:2012 standard using the 4PB-PR method—four-point bending beam. In this method, a prismatic sample is subjected to bending repeatedly with the preservation of free movement and horizontal offset in all pressure and reaction spots. A stiffness modulus |E*| was determined using 48 × 410 × 64 mm (thickness/length/width) samples and calculated in accordance with the following equations:

(5) $$\left|E^{*}\right|=\sqrt{E_{1}{}^{2}+E_{2}{}^{2}}$$

or in a complex version (complex stiffness modulus E*):

(6) $$E^{*}=\left|E^{*}\right|\cdot (\cos\left(\delta \right)+i\cdot \sin(\delta ))$$

and:

(7) $$\delta =\mathrm{arc}\;\tan\left(\frac{E_{2}}{E_{1}}\right)$$

where:

$E_{1}=\left|E^{*}\right|\cdot \cos\left(\delta \right)$—real value of a complex stiffness modulus;

$E_{2}=\left|E^{*}\right|\cdot \sin\left(\delta \right)$—imaginary value of a complex stiffness modulus;

i—an imaginary unit matching the equation $i^{2}=-1$;

$\left|E^{*}\right|$—(dynamic) stiffness modulus—norm (absolute value) of a complex stiffness modulus;

$E^{*}$—a complex stiffness modulus;

δ—a phase angle.

The test was conducted at the temperature of 10 °C. Sinusoidal loading with a frequency of 10 Hz was applied to the sample, which developed the required amplitude of the strain equal to 50 ± 3 μm/m. A stiffness modulus was determined in the hundredth load cycle (it is important for the required parameters, e.g., the amplitude of strain to be constant in the course of a stiffness modulus determination cycle).

### 2.5. Fatigue Resistance Test of Asphalt Mixtures

A fatigue resistance test of an asphalt mixture was conducted in accordance with the EN 12697-24:2018-08 standard on prismatic samples using a four-point bending beam (4PB-PR) method. This method consists in the determination of asphalt mixture sample behaviour after cyclic load in the form of sinusoidal kinematic extortion. Prismatic beams are subjected to bending cyclically providing free rotation and horizontal displacement in every loading and reaction spot. Fatigue resistance was determined for 48 × 410 × 64 mm (thickness/length/width) samples.

Fatigue resistance of an asphalt mixture is specified as the number of loading or strain cycles required to destroy a tested sample (the achievement of conventional fatigue criterion, where the residual value of stiffness modulus decreases by 50% compared to the initial value of stiffness modulus).

The test was conducted at the temperature of 10 °C with load frequency of 10 Hz and with constant strain amplitude equal 130 μm/m. Asphalt mixtures were assessed on the basis of determined value of fatigue damage, which reflects the percentage reduction of stiffness modulus residual value compared to its initial value. An initial value of stiffness modulus was determined after a hundredth load cycle, whereas its residual value was determined after 10^6^ of load cycles.

## 3. Results and Discussion

For each type of examined hot mix asphalt, the authors made: four specimens (for the rutting test); five specimens for the “dry” set and five specimens for the “wet” set (test of resistance to water and frost) and eight specimens (for stiffness modulus and fatigue resistance tests).

**Figure 4:**
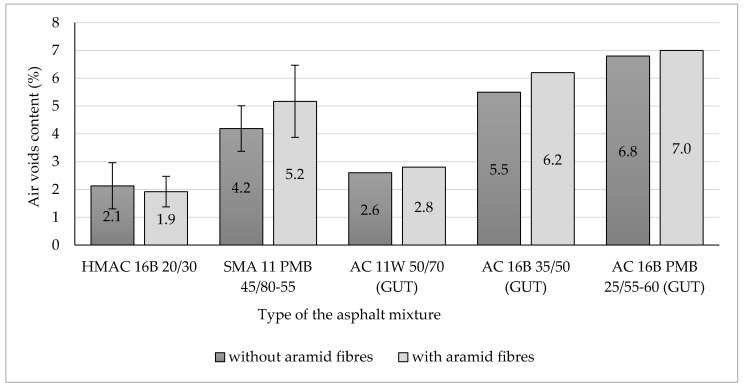
Air voids content of analysed asphalt mixtures (including HMA examined at the Gdańsk University of Technology [26]).

**Figure 5:**
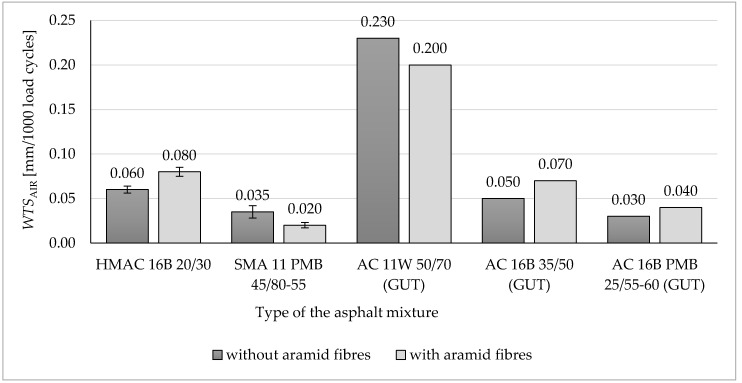
Wheel tracking slope—*WTS*_AIR_ of the analysed HMA (including HMA examined at the Gdańsk University of Technology [26]).

**Figure 6:**
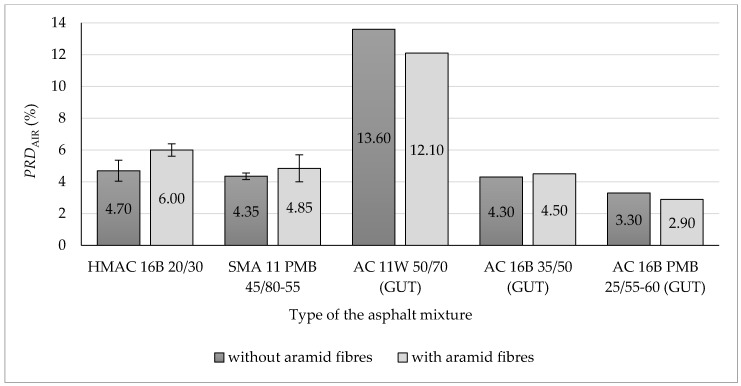
Proportional rut depth—*PRD*_AIR_ after 10,000 load cycles of analysed asphalt mixtures (including HMA examined at the Gdańsk University of Technology [26]).

**Figure 7:**
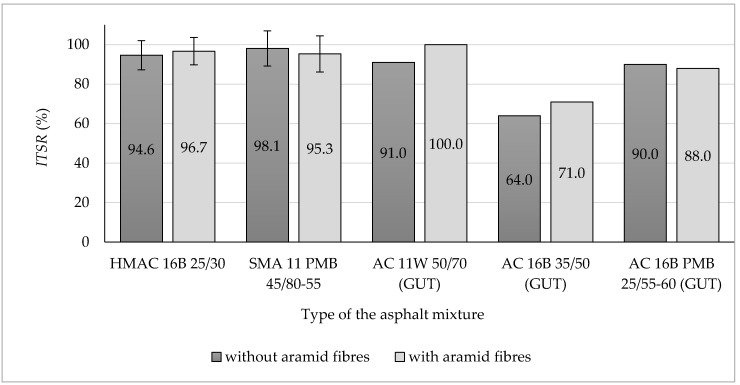
Indirect tensile strength ratio *ITSR* of analysed HMA (including HMA examined at the Gdańsk University of Technology [26]).

**Figure 8:**
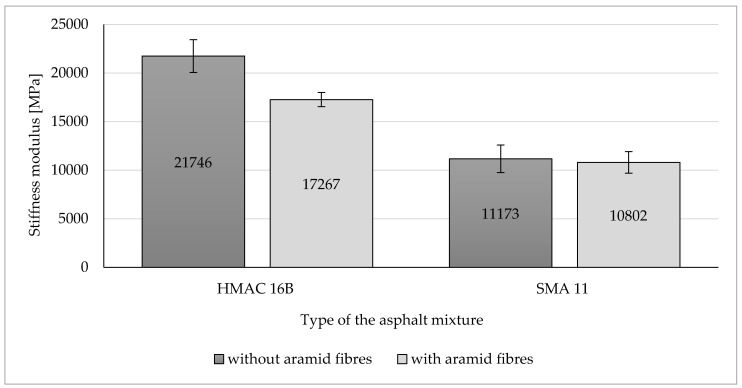
Stiffness modulus values of the analysed HMA.

**Figure 9:**
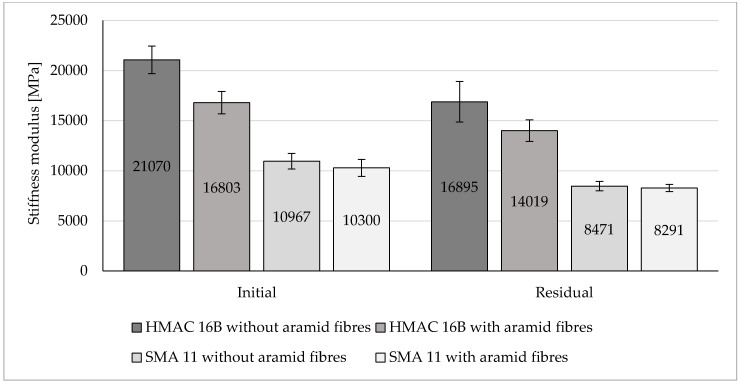
Initial and residual values of stiffness modulus of the analysed HMA.

**Figure 10:**
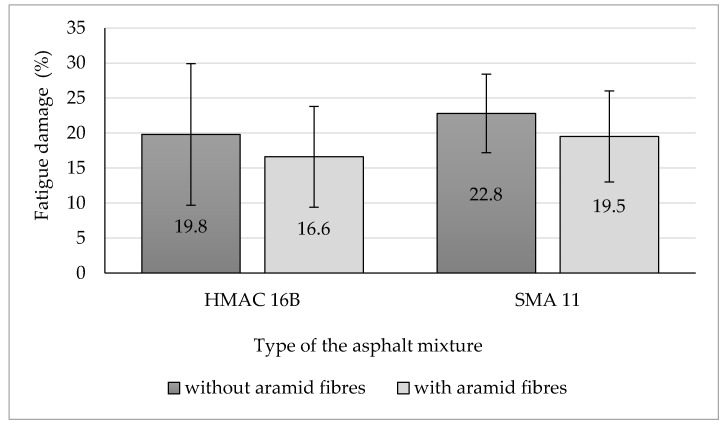
Fatigue damage of analysed asphalt mixtures.

Each set of obtained results was checked by using Grubbs test (performed at the significance level α = 0.05) in order to indicate outliers and remove them from the further analysis. All the results obtained from the research program, showed in Figure 4, Figure 5, Figure 6, Figure 7, Figure 8, Figure 9 and Figure 10, have been presented in the form of mean values (column graphs) and the confidence intervals (error bars) calculated at the significance level α = 0.05 in accordance with the following equation [33]:

(8) $$P\left(\bar{X}-t_{1-\frac{\alpha }{2}}\frac{S}{\sqrt{n}}\right)<µ<P\left(\bar{X}+t_{1-\frac{\alpha }{2}}\frac{S}{\sqrt{n}}\right)=1-\alpha$$

where:

n—number of observations;

$\bar{X}$—sample mean;

S—sample standard deviation;

$t_{1-\frac{\alpha }{2}}$ has a Student’s t-distribution with n − 1 degrees of freedom;

µ—estimated parameter.

Additionally to check, if the differences between parameters was statistically significant, one-way analysis of variance was used as well as Tukey’s range test. Results of this analysis confirmed conclusions formulated from confidence intervals analysis.

### 3.1. Air Void Content in Asphalt Mixtures

The results of air voids content in the tested hot mix asphalt specimens were presented as graph in Figure 4. Additionally, in Figure 4 the results of the air void content for the asphalt mixtures tested at the Gdańsk University of Technology have been included [26].

On the basis of conducted tests, it can be seen that the addition of aramid fibres to the HMAC 16 mixture reduced the mean value of voids by 0.2 percentage points compared to hot mix asphalt without the addition of fibres. The opposite effect was observed for SMA 11, for which the addition of fibres increased the mean value of voids by 1.0 percentage point compared to SMA without aramid fibres.

Taking into consideration confidence intervals differences between mean values of air voids content are not significant from the statistical point of view (at the level of significance α = 0.05) to any of the analysed asphalt mixtures.

Analysing the results obtained by researchers from Gdańsk, it can be observed that aramid fibres addition to the HMA gave similar effect as for SMA 11. The only exception was in increasing in air voids content—it was smaller than for SMA 11.

### 3.2. Resistance of Asphalt Mixtures to Rutting

Results of resistance to rutting tests on analysed asphalt mixtures were presented as graphs in Figure 5 and Figure 6. Values of a wheel-tracking slope *WTS*_AIR_ and proportional rut depth *PRD*_AIR_ have been presented in Figure 5 and Figure 6, respectively. For each type of HMA, the type of asphalt binder used to produce the given asphalt mixture has been presented in the figures. Additionally, in Figure 5 and Figure 6, results of the asphalt mixture tests conducted at the Gdańsk University of Technology have been included [26]. Results received from the Gdańsk University of Technology are marked as GUT on the graphs.

On the basis of conducted tests, it is noticeable that the addition of aramid fibres to the asphalt mixture HMAC 16B resulted in the decrease of resistance to permanent deformations of this mixture. All determined parameters indicate that rutting resistance became worse following aramid fibre addition. A wheel tracking slope mean value (*WTS*_AIR_) for the asphalt mixture with aramid fibres increased by 33% compared to that without fibres (in both cases, requirements included in Polish Technical Requirements WT-2 were fulfilled). Proportional rut depth mean value of the HMAC 16B mixture with the addition of aramid fibres increased by 31% compared to the HMAC 16B mixture without fibres. A proportional rut depth (*PRD*_AIR_) increased following aramid fibre addition to the asphalt mixture—in that case an increase by 28% compared to the asphalt mixture without fibre addition was recorded (requirements concerning *PRD*_AIR_ value included in Polish Technical Requirements WT-2 were fulfilled only for HMAC 16B mixture without fibres). The maximum acceptable values of parameters *WTS*_AIR_ and *PRD*_AIR_ included in Polish Technical Requirements WT-2 regarding HMAC 16B and SMA 11 are presented in Table 4.

A similar impact on rutting resistance was noted after the addition of aramid fibres to stone mastic asphalt (SMA 11). In that case, an increase in rut depth and proportional rut depth *PRD*_AIR_ was also recorded (by 11% compared to SMA without fibres), but the increase of those values compared to asphalt mixtures without fibres was lower than in case of HMAC 16B. The addition of aramid fibres to the SMA mixture had a positive impact only on the *WTS*_AIR_ value, by decreasing a wheel tracking slope mean value by 42% compared to the SMA mixture without fibres. It is worth noting that the analysed SMA 11 mixture in both cases (without fibres and with the addition of aramid fibres) fulfils requirements included in Polish Technical Requirements WT-2.

Taking into consideration confidence intervals differences between mean values of *WTS*_AIR_ are significant from the statistical point of view (at the level of significance α = 0.05) for both analysed asphalt mixtures. Differences between mean values of *PRD*_AIR_ are significant from the statistical point of view only for HMAC 16B.It may be noted after the analysis of tests conducted at the Gdańsk University of Technology (GUT) that the addition of aramid fibres to asphalt concrete AC 16B 35/50 had a negative impact on its resistance to rutting, as it increased *WTS*_AIR_ value, similarly as in the case of HMAC 16B. The proportional rut depth value for AC 16B made of 35/50 penetration grade bitumen increased, as in the case of HMAC 16B. The opposite effect was recorded only for AC 16B PMB 25/55-60 containing polymer modified bitumen, where *PRD*_AIR_ value decreased by 12% (compared to the mixture without fibres) after the addition of aramid fibres.

Another effect of aramid fibre addition to HMA may be noticed for the AC 11W mixture, for which the fibre addition has a positive impact on its resistance to rutting, as it decreases the *WTS*_AIR_ value (by 13% compared to the mixture without fibre addition) and the *PRD*_AIR_ (by 11% compared to the mixture without fibres). It does not apply to SMA 11 because of the increase of *PRD*_AIR_ value.

Comparing results of tests carried out by the authors hereof to results obtained at the Gdańsk University of Technology in terms of rutting resistance, it may be noted that the addition of aramid fibres to HMA designated for a binder course using unmodified road bitumen has a negative impact on HMA rutting resistance. It may not be concluded for AC 16B PMB 25/55-60 containing polymer modified bitumen, as in that case a decrease in *PRD*_AIR_ value was noted.

### 3.3. Resistance of Asphalt Mixtures to Water and Frost

Results of water and frost resistance tests of analysed asphalt mixtures with and without aramid fibres addition have been presented in Figure 7. Moreover, test results obtained at the Gdańsk University of Technology (GUT) have been presented in Figure 7 as well [26].

For analysed asphalt mixtures, both with and without aramid fibre addition, indirect tensile strength ratio (*ITSR*) values fulfilled requirements included in the Polish Technical Requirements WT-2. The addition of aramid fibres increased the asphalt mixture resistance to water and frost only in case of the HMAC 16B mixture, for which the *ITSR* mean value increased by 3.5 percentage points compared to the same mixture without fibre addition. In case of the SMA mixture, the addition of aramid fibres decreased the *ITSR* mean value by 2.1 percentage points compared to the SMA mixture without fibres, which may indicate the reduction of this mixture resistance to water and frost. The minimum acceptable value of parameter *ITSR* included in Polish Technical Requirements WT-2 for analysed hot mix asphalt is presented in Table 5.

Taking into consideration confidence intervals differences between mean values of *ITSR* are not significant from the statistical point of view (at the level of significance α = 0.05) in the case of any of the analysed asphalt mixtures.

In case of HMA tested at the Gdańsk University of Technology (GUT), the addition of aramid fibres improved water and frost resistance for the AC 11W mixture, for which *ITSR* value increased by 9 percentage points compared to the mixture without fibres, which cannot be confirmed for the SMA 11 mixture (the decrease of *ITSR* value was observed after fibre addition). The addition of aramid fibres also had a positive impact on AC 16W asphalt concrete containing 35/50 penetration grade bitumen—the increase of the *ITSR* value by seven percentage points was noted compared to the asphalt mixture without fibres (a positive impact was also observed on HMAC 16B). Another impact may be noticed for AC 16B PMB 25/55-60 containing polymer modified bitumen, for which the addition of fibres decreased the *ITSR* value by two percentage points compared to the reference mixture (AC 16B PMB 25/55-60 without fibres).

It may be noted following the analysis of results of tests carried out by the authors hereof and test results obtained at the Gdańsk University of Technology that the addition of aramid fibres has a positive impact in terms of water and frost resistance only in the case of HMA, which was produced using unmodified road bitumen. In case of polymer modified bitumen, the decrease of *ITSR* value was observed. However, these conclusions refer only to the HMA analysed above.

### 3.4. Stiffness Modulus of Analysed Asphalt Mixtures

Results of stiffness modulus determined for the analysed asphalt mixtures have been presented in Figure 8.

Obtained test results indicates that the addition of aramid fibres to the HMAC mixture decreased a stiffness modulus mean value by 20% compared to the value for that mixture without fibres. On the other hand, no significant change in stiffness modulus mean value was observed following the addition of aramid fibres to the SMA mixture (decrease by 3% compared to the SMA mixture without fibres).

Taking into consideration confidence intervals differences between mean values of stiffness modulus are significant from the statistical point of view (at the level of significance α = 0.05) only for HMAC 16B.

### 3.5. Fatigue Resistance of Analysed Asphalt Mixtures

Initial and residual values of stiffness modulus have been presented in Figure 9. Values of fatigue damage characterizing fatigue resistance of the examined HMA, have been presented in Figure 10. Fatigue damage has been described as a proportional decrease of the residual value of stiffness modulus (after 10^6^ load cycles) compared to its initial value (after 10^2^ load cycles) using the following equation:

(9) $$D=\frac{\left|E_{i}^{*}\right|-\left|E_{r}^{*}\right|}{\left|E_{i}^{*}\right|}\cdot 100\;\left(\%\right)$$

where:

D—fatigue damage (%);

$\left|E_{r}^{*}\right|$—a residual value of stiffness modulus after 10^6^ load cycles (MPa);

$\left|E_{i}^{*}\right|$—an initial value of stiffness modulus after 10^2^ load cycles (MPa).

The addition of aramid fibres decreased residual values of stiffness modulus in case of both analysed types of asphalt mixtures.

The analysis of fatigue damage values indicate that the addition of aramid fibres have positive impact on fatigue resistance of the analysed asphalt mixtures. In case of the HMAC 16B mixture, a fatigue damage mean value decreased by 3.2 percentage points following the addition of aramid fibres compared to the mixture without fibres. Similar impact on fatigue resistance has been noticed in the case of SMA mixture, for which the addition of aramid fibres decreased the fatigue damage mean value by 3.3 percentage points (compared to the SMA mixture without fibres). On that basis, one may conclude that the addition of aramid fibres to HMAC and SMA mixtures improves their fatigue resistance. However, taking into consideration confidence intervals, the observed differences concerning determined mean values of fatigue damage are not significant from the statistical point of view (at the level of significance α = 0.05).

## 4. Conclusions

Based on the results of conducted tests, the following findings may be presented and observations made:

(a) Aramid fibres addition to the HMAC 16B slightly reduced the mean air voids content. The opposite effect was observed for the SMA 11—the addition of aramid fibres increased the mean air void content.
(b) The addition of aramid fibres did not improve resistance to permanent deformation (rutting) of analysed asphalt mixtures. In case of both analysed asphalt mixtures, this addition caused an insignificant decrease of all determined parameters characterising rutting resistance, with the exception of wheel tracking slope value for SMA 11 (in that case, the *WTS*_AIR_ decreased insignificantly). These observations confirm results of rutting tests of different types of HMA obtained at the Gdańsk University of Technology. It was observed that the addition of aramid fibres decreased also rutting resistance in case of AC 16B 35/50, similarly as it was in case of HMAC 16B and SMA 11 mixtures. The positive impact on rutting resistance was observed in case of AC 11W and AC 16B PMB 25/55-60 mixtures investigated at the Gdańsk University of Technology.
(c) The impact of aramid fibre addition on the asphalt mixture resistance to water and frost varies for analysed mixtures. In the case of the HMAC 16B mixture, the addition of fibres resulted in an insignificant increase of an *ITSR* value, whereas in the case of the SMA mixture a small decrease of *ITSR* value was observed compared to referential mixtures (without fibres). Comparing to test results obtained at the Gdańsk University of Technology, it was observed that the *ITSR* value increased following the addition of aramid fibres to AC 11W and AC 16B 35/50 mixtures, which may indicate the improvement of resistance to water and frost only in case of mixtures containing penetration grade bitumens (unmodified). In case of mixtures containing polymer modified bitumens, *ITSR* values were reduced, which is indicated both by test results obtained at the Gdańsk University of Technology (AC 16B PMB 25/55-60) and by results obtained by authors hereof (SMA 11 PMB 45/80-55). However, this statement does not refer to all types of HMA due to the lack of results referring to other types of HMA.
(d) The addition of aramid fibres decreased the stiffness modulus mean values of HMAC 16B mixture. In case of SMA 11 mixture, no significant differences between stiffness modulus values for the reference mixture and the mixture with the addition of aramid fibres were noticed (difference was only three percentage points), which does not enable to explicitly specify the impact of aramid fibre addition on stiffness modulus of an asphalt mixture.
(e) A fatigue damage mean values characterising fatigue resistance in both cases of analysed asphalt mixtures (HMAC 16B and SMA 11) with the addition of aramid fibres, decreased compared to HMAC asphalt mixtures without fibres. It may indicate an improvement in fatigue resistance of both asphalt mixtures.

On the basis of the obtained test results, it is difficult to state explicitly what the impact of aramid fibre addition is on the analysed asphalt mixtures. The content of 0.05% (compared to the mass of asphalt mixture) recommended by the producer may be too low to cause a significant improvement of functional properties of an examined HMA. More promising results were obtained from tests carried out at the Gdańsk University of Technology, which may indicate that the impact of aramid fibre addition may be different for various types of asphalt mixtures.

The future direction of research will include the assessment of HMA properties influenced by aramid fibres in greater amounts than recommended by the producer and tests of other asphalt mixture types.

## Figures and Tables

**Table 1:** Results of penetration and softening point determination of asphalt binders used in the production of analysed asphalt mixtures.

Type of Bitumen	Penetration at 25 °C (mm/10)	Softening Point (°C)
20/30	23.5	58.6
PMB 45/80-55	62.0	67.4

**Table 2:** Content of components of the HMAC 16B mixture with and without aramid fibres.

Name of the Mixture Component	Content in the Mixture (%)			
	Without Fibres		With Aramid Fibres	
	AM	HMA	AM	HMA
limestone filler	5.5	5.21	5.5	5.21
fine amphibolitic aggregate 0/2	35.0	33.18	35.0	33.15
coarse amphibolitic aggregate (grit) 2/5	20.0	18.96	20.0	18.95
coarse amphibolitic aggregate (grit) 5/8	10.5	9.96	10.5	9.96
coarse amphibolitic aggregate (grit) 8/11	11.0	10.43	11.0	10.43
coarse amphibolitic aggregate (grit) 11/16	18.0	17.06	18.0	17.05
20/30 penetration grade bitumen	-	5.20	-	5.20
aramid fibres	-	-	-	0.05

**Table 3:** Content of components of the SMA 11 mixture with and without aramid fibres.

Name of the Mixture Component	Content in the Mixture (%)			
	Without Fibres		With Aramid Fibres	
	AM	HMA	AM	HMA
limestone filler	9.0	8.44	9.0	8.44
fine amphibolitic aggregate 0/2	15.0	14.07	15.0	14.06
coarse amphibolitic aggregate (grit) 2/5	15.0	14.07	15.0	14.06
coarse amphibolitic aggregate (grit) 5/8	12.0	11.26	12.0	11.26
coarse amphibolitic aggregate (grit) 8/11	49.0	45.96	49.0	45.93
PMB 45/80-55 polymer modified bitumen	-	6.20	-	6.20
aramid fibres	-	-	-	0.05

**Table 4:** Polish Technical Requirements WT-2 for the rutting resistance of HMAC 16B and SMA 11.

Type of the Asphalt Mixtures	*WTS*_AIR_ (mm/10^3^ Load Cycles)	*PRD*_AIR_ (%)
HMAC 16B	≤0.10	≤5.0
SMA 11	≤0.15	≤7.0

**Table 5:** Polish Technical Requirements WT-2 for the resistance of asphalt mixtures to water and frost for HMAC 16B and SMA 11.

Type of the Asphalt Mixtures	*ITSR* (%)
HMAC 16B	≥80
SMA 11	≥90
